# Editorial: Plant-based foods: harmonizing health promotion and sustainability strategies from a high-throughput perspective

**DOI:** 10.3389/fnut.2026.1766368

**Published:** 2026-01-21

**Authors:** Begoña Miras-Moreno, Leilei Zhang, Pascual García-Pérez

**Affiliations:** 1 Department of Plant Biology, Faculty of Biology, University of Murcia, Campus de Espinardo, Murcia, Spain; 2 Department for Sustainable Food Process, Università Cattolica del Sacro Cuore, Piacenza, Italy; 3 Department of Food Technology, Nutrition and Food Science, Veterinary Faculty, University of Murcia, Regional Campus of International Excellence “Campus Mare Nostrum”, Murcia, Spain

**Keywords:** nutraceuticals, nutrition, omics, One Health, plant-based

The Topic Collection titled “*Plant-Based Foods: Harmonizing Health Promotion and Sustainability Strategies from a High-Throughput Perspective*” provided crucial insights across health outcomes, sustainable production methods, specific nutritional challenges, and consumer behavior in the field of plant-based foods. The five contributions making part of the Research Topic establish a high-throughput perspective on the nutritional and sustainable future of plant-based diets, meeting the goals established during the conception of the topic. The four main key points covered in this topic conferred an updated and a multidisciplinary assessment in the study of plant-based foods and the establishment of sustainable agri-food systems.

First, the contribution by Tan et al., titled “*Plant-based diet and risk of all-cause mortality: a systematic review and meta-analysis*” represented the first milestone on health promotion and diet quality. The fundamental objective of promoting plant-based diets for human health is strongly validated, but with a critical distinction based on food choices, according to the systematic review and meta-analysis. This study further confirmed that the association between a plant-based diet and mortality is dependent on the quality of the food consumed. Owing to the results, the adherence to a Healthy Plant-Based Diet Index (hPDI) was significantly linked to a reduction in overall mortality (Relative Risk (RR) = 0.85, 95% CI 0.80–0.90) and cardiovascular disease (CVD) mortality (RR = 0.85, 95% CI 0.77–0.94). The hPDI emphasizes healthy plant-based foods such as whole grains, nuts, vegetables, legumes, coffee, and tea. In contrast, greater adherence to an Unhealthy Plant-Based Diet Index (uPDI) is associated with an increased risk of overall mortality (RR = 1.18, 95% CI 1.09–1.27) and CVD mortality (RR = 1.19, 95% CI 1.07–1.32). In this case, the uPDI includes less healthy plant-based options like refined grains, potatoes, fruit juices, and sugary drinks. Overall, the preventive effect of hPDI appears to be more pronounced in individuals over 55 years old, providing insight into the real-world assessment of dietary quality of plant-based diets in human health.

In parallel, Hetting et al. featured a timely review titled “*Plant-based proteins for infant formula: findings and recommendations from the ILSI Europe workshop*” focused on the main challenges of transitioning to plant-based systems for vulnerable populations, claiming about the establishment of rigorous safety and quality assessment in children nutrition. Their findings showed that in the European Union (EU), only soy protein isolate and hydrolyzed rice protein isolate are currently authorized for infant formulas, urging the incorporation of emerging sources like pea, lentil, and faba bean. These protein sources are promising candidates due to their protein content, availability, and sustainability benefits (nitrogen fixation). However, the authors claim that Antinutritional Factors (ANFs) represent significant hurdles reducing protein digestibility and the bioavailability of essential nutrients. To overcome current limitations in childhood nutrition, these researchers recommend accelerating regulatory approval by focusing on a few promising candidates, building robust and sustainable extraction processes, developing reliable *in vitro* models of the infant gut, and conducting clinical studies to confirm safe growth and development outcomes.

In another research article, Wang et al. explored the consumer behavior and sustainable eating in vegetarian restaurants aiming at the establishment of sustainable tourism paradigm. The publication, titled “*Impact of vegetarian restaurant experiences on pro-environmental eating behaviors: a sustainable tourism perspective*,” is committed to maximize the impact of plant-based foods through the influence of consumer behaviors. Their research is based on the application of the Stimulus-Organism-Response (SOR) framework in vegetarian restaurants, identifying that external stimuli drove internal psychological processes that resulted in pro-environmental behaviors. Specifically, the external stimuli—sensory experience (e.g., ambiance, taste, sound) and perceived green knowledge (e.g., knowing the environmental benefits)—positively and significantly enhanced the internal cognitive states, resulting in a positive impact on environmentally friendly dietary behavior, such as minimizing food waste. The findings highlighted that communication strategies should link sensory pleasure with environmental and health knowledge to foster deeper, reflective behavioral changes.

Meshram et al. contributed with a timely article titled “*New Insights into Organic Agriculture: Innovations, Challenges, and Opportunities*” to emphasizes the importance of food production systems in addressing environmental challenges within the broader scope of plant-based foods. These authors defended that the drive toward sustainability in food production requires both shifts in farming models and innovative technological inputs. Organic Agriculture (OA) models, and the feasibility of Synchronized Nutrition Fertilizer (SNFert) are proposed as the main drivers to optimize soil health while matching sugarcane nutrient release to crop demands and reducing chemical inputs, thus creating suitable conditions for beneficial soil microbes.

The collective evidence underscores that the promotion and scaling of plant-based foods must integrate rigorous health and nutrition validation, sustainable agricultural innovation, and effective behavioral communication strategies to achieve the goal of a harmonious, high-throughput food system. In this sense, to summarize the scope of our Research Topic, the shift toward sustainable food systems requires a complex, multi-faceted approach that integrates stringent quality control, technological innovation, and consumer strategies.

